# Icariin Ameliorates Cigarette Smoke Induced Inflammatory Responses via Suppression of NF-κB and Modulation of GR *In Vivo* and *In Vitro*

**DOI:** 10.1371/journal.pone.0102345

**Published:** 2014-08-04

**Authors:** Lulu Li, Jing Sun, Changqing Xu, Hongying Zhang, Jinfeng Wu, Baojun Liu, Jingcheng Dong

**Affiliations:** 1 Department of Integrated Traditional Chinese and Western Medicine, Huashan Hospital, Fudan University, Shanghai, China; 2 Hangzhou Normal University, School of medicine, Affiliated Hospital, Hangzhou, China; National Institutes of Health, United States of America

## Abstract

**Purpose:**

To investigate the effects of icariin, a major constituent of flavonoids isolated from the herb *Epimedium*, on cigarette smoke (CS) induced inflammatory responses *in vivo* and *in vitro*.

**Methods:**

*In vivo*, BALB/c mice were exposed to smoke of 15 cigarettes for 1 h/day, 6 days/week for 3 months and dosed with icariin (25, 50 and 100 mg/kg) or dexamethasone (1 mg/kg). *In vitro*, A549 cells were incubated with icariin (10, 50 and 100 µM) followed by treatments with CSE (2.5%).

**Results:**

We found that icariin significantly protected pulmonary function and attenuated CS-induced inflammatory response by decreasing inflammatory cells and production of TNF-α, IL-8 and MMP-9 in both the serum and BALF of CS-exposed mice and decreasing production of TNF-α and IL-8 in the supernatant of CSE-exposed A549 cells. Icariin also showed properties in inhibiting the phosphorylation of NF-κB p65 protein and blocking the degradation of IΚB-α protein. Further studies revealed that icariin administration markedly restore CS-reduced GR protein and mRNA expression, which might subsequently contribute to the attenuation of CS-induced respiratory inflammatory response.

**Conclusion:**

Together these results suggest that icariin has anti-inflammatory effects in cigarette smoke induced inflammatory models *in vivo* and *in vitro*, possibly achieved by suppressing NF-κB activation and modulating GR protein expression.

## Introduction

Chronic obstructive pulmonary disease (COPD) is a chronic inflammatory disease that is characterized by airway obstruction and progressive lung inflammation that is associated with the influx of inflammatory cells such as neutrophils, macrophages, lymphocytes and epithelial cells [1], [2], as well as increases in the levels of a complex cascade of inflammatory mediators including chemokines such as IL-8 and pro-inflammatory cytokines such as TNF-α, IL-1β, IL-6 and MMP-9 [3], [4]. It is predicted by the World Health Organization that COPD will become the fifth most common cause of morbidity and the third most common chronic disease worldwide [5], [6].

As a chronic inflammatory disease, COPD responds poorly to currently available therapies, particularly corticosteroids, which are the most effective anti-inflammatory approaches available for a wide range of chronic inflammatory diseases, including asthma, rheumatoid arthritis and inflammatory bowel disease [7], suggesting that the inflammation in COPD is essentially corticosteroid resistant [8], [9]. The molecular mechanisms that underlying the inflammation with glucocorticoid insensitivity in COPD are currently still unclear, but a possible explanation may involve the impaired ability of glucocorticoid receptor (GR) [10], which mediates the effect of glucocorticoid by translocating into the nucleus, binding to glucocorticoid responsive elements (GRE) in the DNA or with the transcription factors activating protein-1 (AP-1) and nuclear factor-κB (NF-κB), thereby preventing inflammatory gene expression [11], [12]. Therefore, a decrease in GR may lead to a decrease in complex formation, increasing the free NF-κB in the nucleus to bind to the DNA. This could be seen as an early response to cigarette smoke in the lungs with direct access of NF-κB to the opened promoters [13]. In view of the inflammation with glucocorticoid insensitivity in COPD, there is a pressing need for the development of effective anti-inflammatory drugs.

**Figure 1:**
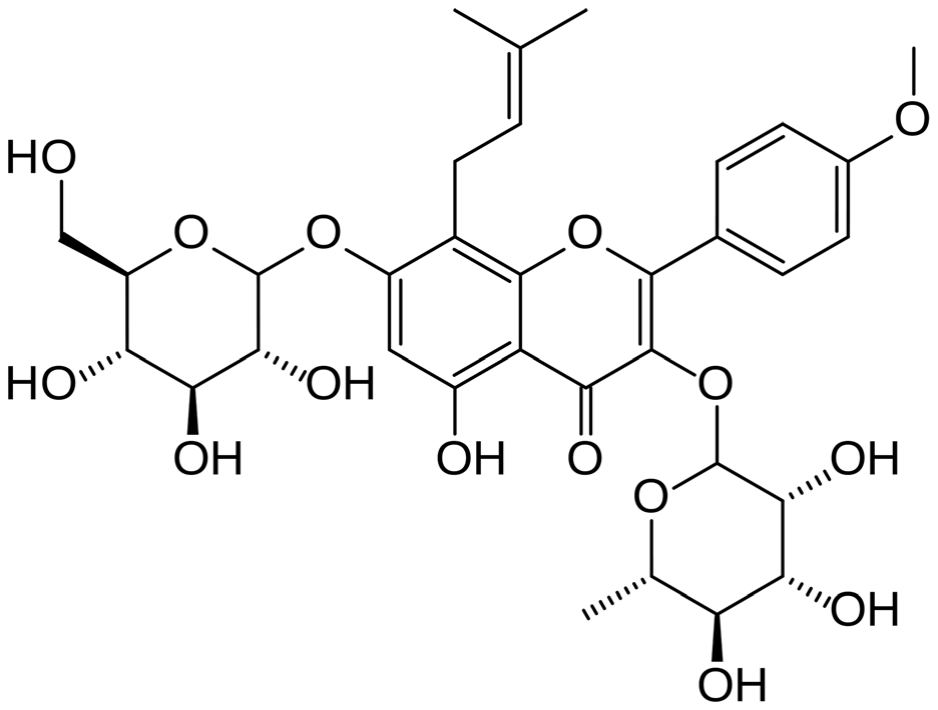
Chemical structure of icariin. (molecular formula: C_33_H_40_O_15_; molecular weight: 676.67).

Icariin (Fig. 1), a major constituent of flavonoids isolated from the herb *Epimedium*, has been confirmed to exert the effects of anti-inflammation, anticancer, cardiovascular protection and bone formation promotion [14], [15]. Our studies have demonstrated that icariin and its derivative, 3,5,7-trihydroxy-4′-methoxy-8- (3-hydroxy-3-methylbutyl)-flavone(ICT), attenuates lipopolysaccharide (LPS)- induced inflammatory response mouse models of inflammation and in murine macrophage cell lines, which involve down-regulation of IL-6 and TNF-α, as well as activation of the PI3K/Akt pathway and inhibition of NF-κB [16], [17]. Recently we reported that icariin attenuated social defeat-induced down-regulation of glucocorticoid receptor in a mouse model for depression by enhancing GR binding affinity and protein expression [18].

The aim of this study was to perform a comprehensive assessment of the role of icariin in the inflammatory response associated with CS exposure and the development of COPD. In the present study, we first investigated the anti-inflammatory potential of icariin and further studied whether icariin suppress NF-κB and modulate GR in CS-induced lung inflammation, using BALB/c mice and A549 epithelial cells as animals and cells models of COPD respectively. This study might provide insight into an application of icariin on COPD, and lead to the development of new effectively anti-inflammatory drugs in COPD.

## Materials and Methods

### Materials

Icariin was purchased from Shanghai Ronghe Corporation (Shanghai, China). Peony brand cigarettes were purchased from Shanghai Tobacco Corporation (Shanghai, China). Dexamethasone-Water Soluble (D-2915) was supplied by Sigma-Aldrich (St Louis, MO, USA). Rabbit polyclonal anti GR antibody (sc-8992) was procured from Santa Cruz Biotechnology Inc. (Santa Cruz, CA, USA). Polyclonal anti NF-κB P65 antibody(#3987), polyclonal anti phosphor-P65 antibody (#3039) and anti IκB-α antibody (#9242) were purchased from Cell Signaling Technology (CST, MA, USA).

### Animal studies

Male BALB/c mice (6 weeks old) purchased from Shanghai SLAC Corporation (Shanghai, China) were housed at a constant temperature (23°C) with a 12 h light/dark cycle (lights on from 7:00 A.M. to 7:00 P.M.) and had free access to water and food. All animals were handled in strict accordance with good animal practice as defined by the relevant national and local animal welfare bodies, and all animal work was approved by the Institutional Review Board of Shanghai Medical College of Fudan University (permit number: SYXK(hu)2010–0099), and in accordance with the guidelines for animal use of National Institutes of Health.

Mice (n = 10 per group) were exposed whole body to the smoke of 15 cigarettes (1.0 mg of nicotine and 13 mg of tar per cigarette) for 1 h/day, 6 days/week for 3 months [19], [20] and dosed with icariin (25 mg/kg, 50 mg/kg, 100 mg/kg) or dexamethasone (1 mg/kg) by intragastric administration in 0.3 ml volume 1 hour before exposure to cigarette smoke (CS). Air-exposed mice (control group) underwent none of the procedures. Twenty-four hours after the last exposure, the mice were sacrificed by cervical dislocation and bronchoalveolar lavage (BALF), serum, and lung tissue were obtained. After lung mechanics measurement, the serum and BALF were separated by centrifuging at 1500 g for 10 min at 4°C, and stored at −80°C until the assays were performed.

### Pulmonary function

Basal lung function was measured in unrestrained mice using a whole-body plethysmography (WBP) system (Buxco Electronics, Wilmington, NC). The use of this method permitted continuous monitoring of a number of ventilatory parameters, including peak expiratory flow (PEF), peak inspiratory flow (PIF) and minute ventilation (MV).

### Bronchoalveolar Lavage Fluid

Animals were anesthetized with intraperitoneal pentobarbital (40 mg/kg) The lungs were then lavaged by inserting a cannula into the trachea with 4×0.3 ml aliquots of ice-cold sterile phosphate-buffered saline (PBS). All aliquots were combined for individual mice. The bronchoalveolar lavage fluid (BALF) was immediately centrifuged at 1500 g for 10 min at 4°C, and the cell-free supernatant was stored at −80°C.

### Pulmonary Histopathology

Lungs were fixed in 10% paraformaldehyde, embedded in paraffin, and stained with hematoxylin and eosin (HE). Histopathological assessment of the bronchus and the parenchyma was performed in a blind fashion. The severity of inflammation was scored on a 0–3 scale defined as: 0 =  no inflammatory response; 1 =  mild inflammation with foci of inflammatory cells in bronchial or vascular wall and in alveolar septa; 2 =  moderate inflammation with patchy inflammation or localized inflammation in the walls of the bronchi or blood vessels and alveolar septa, and involvement of less than one-third of the total cross-sectional area of the lung; and 3 =  severe inflammation with diffuse inflammatory cells in the walls of the bronchi or blood vessels and alveoli septa with involvement of one-third to two-thirds of the lung area [21].

The change of air space size was assessed using a modification of the procedure used by Sato et al [22] of determining the mean linear intercepts (MLI). Ten randomly selected fields in each section at ×100 magnification were used and numbers of alveolar septum in the area were calculated. The destructive index (DI) was calculated to evaluate the destruction of the alveolar wall according to the method described by Saetta et al [23]. Ten randomly selected fields in each section at ×50 magnification were utilized to measure DI.

### Preparation of aqueous cigarette smoke extract (CSE)

Aqueous cigarette smoke extract (CSE) was used to mimic the effects of cigarette smoke *in vitro*. One unfiltered cigarette was combusted with the use of a peristaltic pump and cigarette smoke was slowly bubbled into 10 ml culture medium [24], [25], [26]. The pH of the medium- contained smoke extract was adjusted to 7.4 and filter-sterilized through a 0.22 µm syringe filter in order to remove bacteria and large particles. This preparation was considered as 10% CSE concentration and applied for the experiment within 30 minutes.

### Cell Culture and Treatments

A549 cells (human type II alveolar epithelial carcinoma cell lines) were obtained from Chinese Academy of Sciences (Shanghai, China) and maintained in DMEM high glucose culture medium supplemented with 10% fetal bovine serum (FBS), 100 U/ml penicillin and 100 µg/ml streptomycin at 37°C in a humidified atmosphere with 5% CO_2_. All cell culture reagents were purchased from HyClone Laboratories (Logan, UT, USA).

A549 cells were grown to 70–80% confluency in 100 mm culture plates in a total volume of 5 ml DMEM medium containing 10% FBS. After serum-starved for 12 h and washed with ice-cold sterile PBS, cells were treated with CSE (2.5%). For treatments with icariin (1 µM, 10 µM, 50 µM), cells were incubated for 2 h, followed by the addition of CSE for a further 24 h.

### Cytokine Analysis

Interleukin (IL)-8, tumor necrosis factor-α (TNF-α), and matrix metalloproteinase (MMP-9) levels in both BALF and serum, and IL-8 and TNF-α levels in A549 cells culture supernatant were measured using ELISA kits (Quantikine; R&D Systems, Minneapolis, MN) according to the protocol of the manufacturer.

### Immunohistochemical Analysis

Immunohistochemistry (IHC) stain was performed using a two-step EnVision/HRP technique (Dako Cytomation, Denmark) according to the manufacturer's instruction. Polyclonal rabbit anti GR antibody (sc-8992) (dilution 1∶100) was procured from Santa Cruz Biotechnology Inc. (Santa Cruz, CA, USA). The omission of primary antibodies was used as the negative control. Three slides were read at 10 fields of high power (400×) and cytoplasm stained with brown was scored as positive. The expression of GR was quantitatively evaluated using an Olympus BH2 microscope with computer- aided images analysis system (Qiu Wei Inc, Shanghai, China). The digital images were archived by a digital camera (Nikon 4500, Tokyo, Japan). The positive area and optical density (OD) of GR-positive cells were determined by measuring three randomly selected microscopic fields (25×10) for each slide. The IHC index was defined as average integral optical density (AIOD) (AIOD  =  positive area × OD/total area).

### Western Blotting

Lung tissue samples were homogenized and the nuclear proteins were extracted respectively according to the instructions of a nuclear protein extraction kit (Beyotime Biotechnology, China). Protein concentrations were determined using a BCA protein assay kit. For Western blots, equal amounts of protein were separated by 8% SDS-polyacrylamide gel electrophoresis, and transferred to polyvinylidene difluoride (PVDF) membranes. The membranes were then blocked at room temperature for 1.5 h with 5% (w/v) non-fat milk in TBST buffer and incubated with the primary antibodies in TBST overnight at 4°C with continuous shaking. After three washes in TBST, membranes were incubated with secondary antibodies conjugated with horseradish peroxidase for 1 h and visualized using enhanced chemiluminescence ECL kit. The P65, P-P65 IκB-α and GR bands intensities were adjusted with reference to GAPDH control intensities.

### Real-time polymerase chain reaction

Total RNA was isolated from A549 cells using Trizol reagent (Invitrogen, CA, USA) according to the manufacturer's instructions. The first strand cDNA was then synthesized from 2 µg of RNA of each sample using a reverse transcription system (Promega, WI, USA) for real time PCR. PCR mixture was prepared using SYBR Green Realtime PCR Master Mix (Toyobo, Osaka, Japan) and using the primers as follows: for GR, 5′- CCAACGGTGGCAATGTGAAAT -3′ (forward) and 5′-CCAAGGACTCTCATTCGTCTCTT-3′ (reverse); for β-actin, 5′- GCTCTTTTCCAGCCTTCCTT -3′ (forward) and 5′- TGATCCACATCTGCTGGAAG-3′ (reverse). PCR was conducted using the following parameters: an initial denaturation at 95°C for 5 min, followed by 25 cycles of amplification (95°C for 30 s, 60°C for 40 s, and 72°C for 1 min) with a final extension step at 72°C for 7 min. The fluorescence signal was detected at the end of each cycle. The GR mRNA expression was normalized against β-actin and gene expression changes induced by various treatments were determined by the 2^−△△CT^ method [27].

### Statistical Analysis

Data are expressed as mean ± SEM. Statistically significant differences between groups were determined by ANOVA followed by Bonferroni's post-hoc comparisons tests. All analyses were undertaken using the statistical software SPSS 18.0. A value of P<0.05 was considered statistically significant.

## Results

### Effects of icariin on pulmonary function in CS-exposed mice

**Figure 2:**
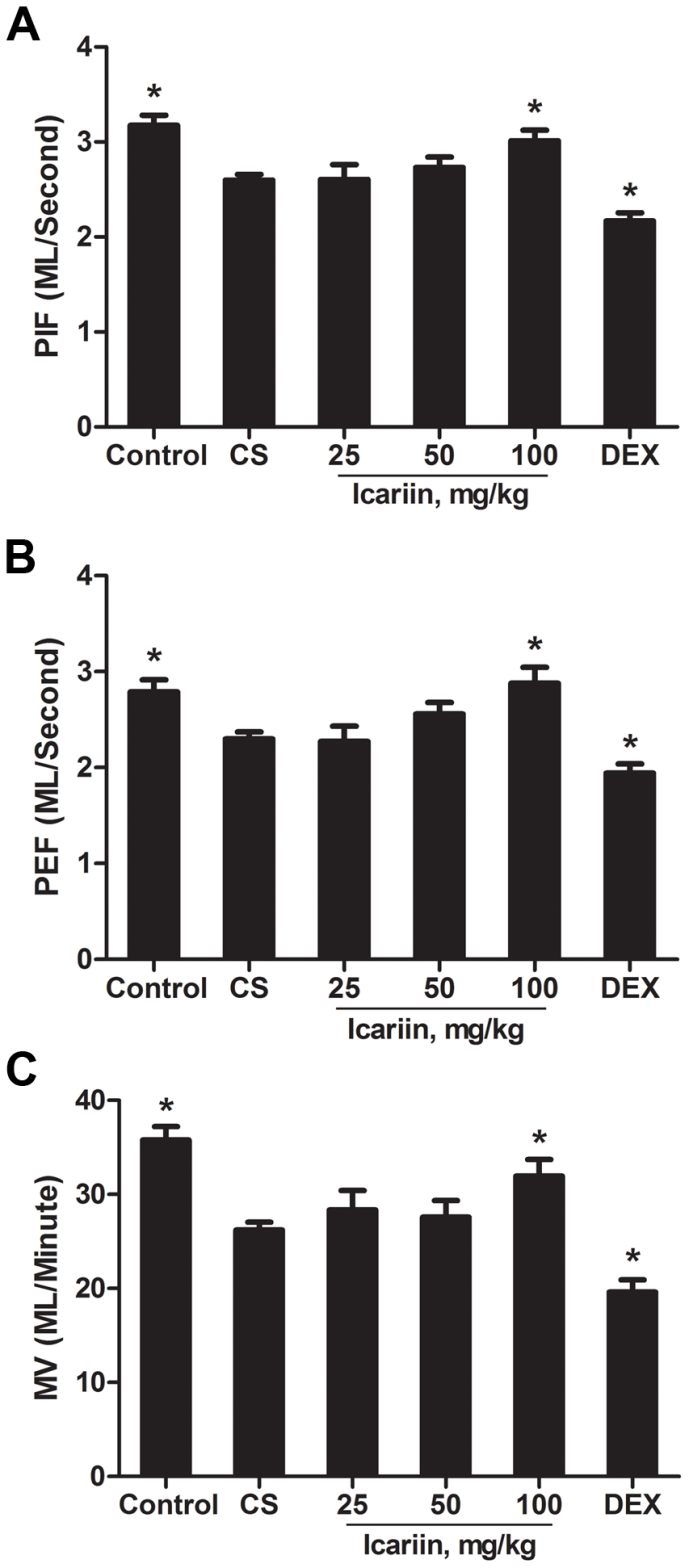
Effects of icariin on pulmonary function in CS-exposed mice. (A) Peak expiratory flow (PEF); (B) Peak inspiratory flow (PIF); (C) Minute ventilation (MV), were measured in unrestrained mice using a whole-body plethysmograph (WBP) system. Data are mean ± SEM (n = 10). * P<0.05 vs. CS-exposed mice. DEX = dexamethasone.

To examine whether icariin restored pulmonary function in CS-exposed mice, breathing parameters were determined by whole-body barometric plethysmography after 3 months of exposure to air or cigarette smoke. CS-exposed mice demonstrated marked and consistent decreases in peak inspiratory flow (PEF), peak expiratory flow (PIF) and minute ventilation (MV) whereas mice treated with high dose of icariin showed significantly (P<0.05) growth on these breathing parameters compared with CS-exposed mice (Fig. 2). Unlike in the icariin groups, the pulmonary function in the dexamethasone group was even significantly lower than that in CS-exposed group, which may confirm the insensitivity to glucocorticoid described as before.

### Effects of icariin on lung histopathology in mice

**Figure 3:**
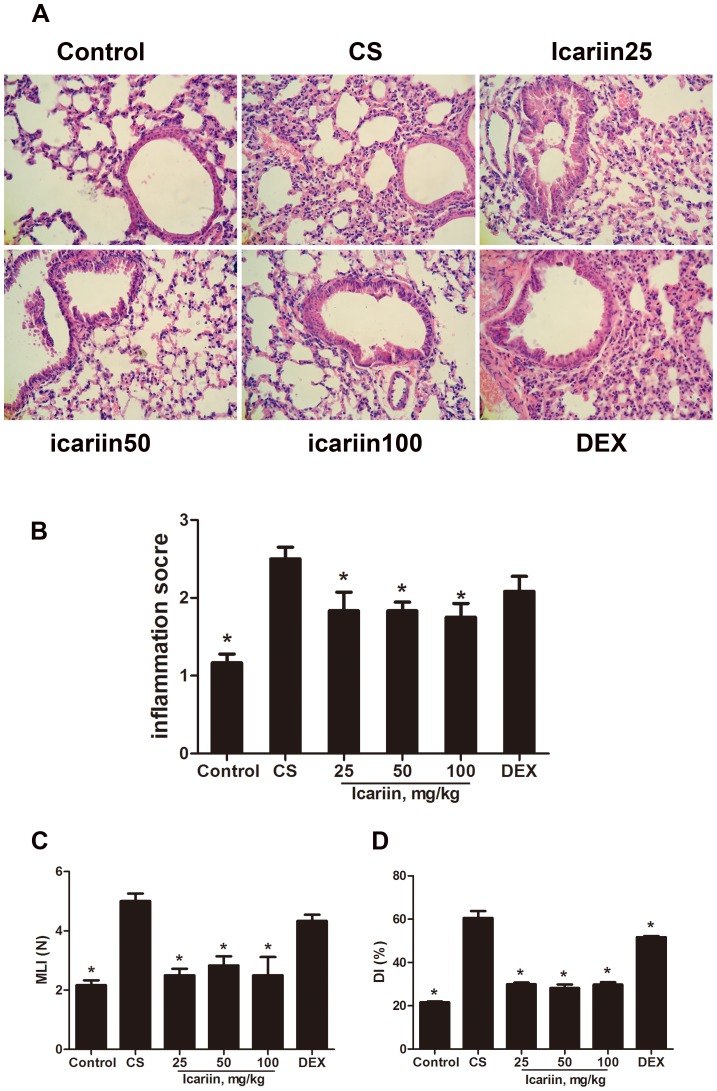
Effects of icariin on lung histopathology. Mice were exposed to CS for 3 months and treated with icariin (25, 50 and 100 mg/kg) or dexamethasone (1 mg/kg). Lung tissue was stained by H&E before being examined. (A) The inflammatory cells of lungs measured by bright microscopy (original magnification,×400). (B) The severity of inflammation was calculated on a 0–3 scale defined as described in Materials and methods. Values of MLI (C) and DI (D) were scored also as described in Materials and methods. Data are mean ± SEM (n = 6). *P<0.05 vs. CS-exposed mice. DEX =  dexamethasone; MLI =  mean linear intercepts; DI =  destructive index.

Lung tissue was stained using H&E before being examined. We found that (Fig. 3 A, B) there were significant histopathological changes in the airway and the lung parenchyma of CS-exposed mice, including interstitial edema, massive infiltration of the inflammatory cells and pulmonary architecture damage, as compared as controls. Icariin (50 mg/kg and 100 mg/kg) significantly decreased the inflammatory cells in response to CS. However, this effect was not evident in mice treated with dexamethasone.

In addition, our data showed that 3 doses of icariin effectively (P<0.05) reduced both the mean linear intercepts (MLI) and the destructive index (DI), which were highly increased in CS-exposed mice as compared as controls. In comparison, mice treated with dexamethasone did not display a significantly (P>0.05) different MLI from that observed in CS-exposed mice (Fig. 3C, D).

### Effects of icariin on CS-induced TNF-α, IL-8 and MMP-9 levels in vivo and in vitro

**Figure 4:**
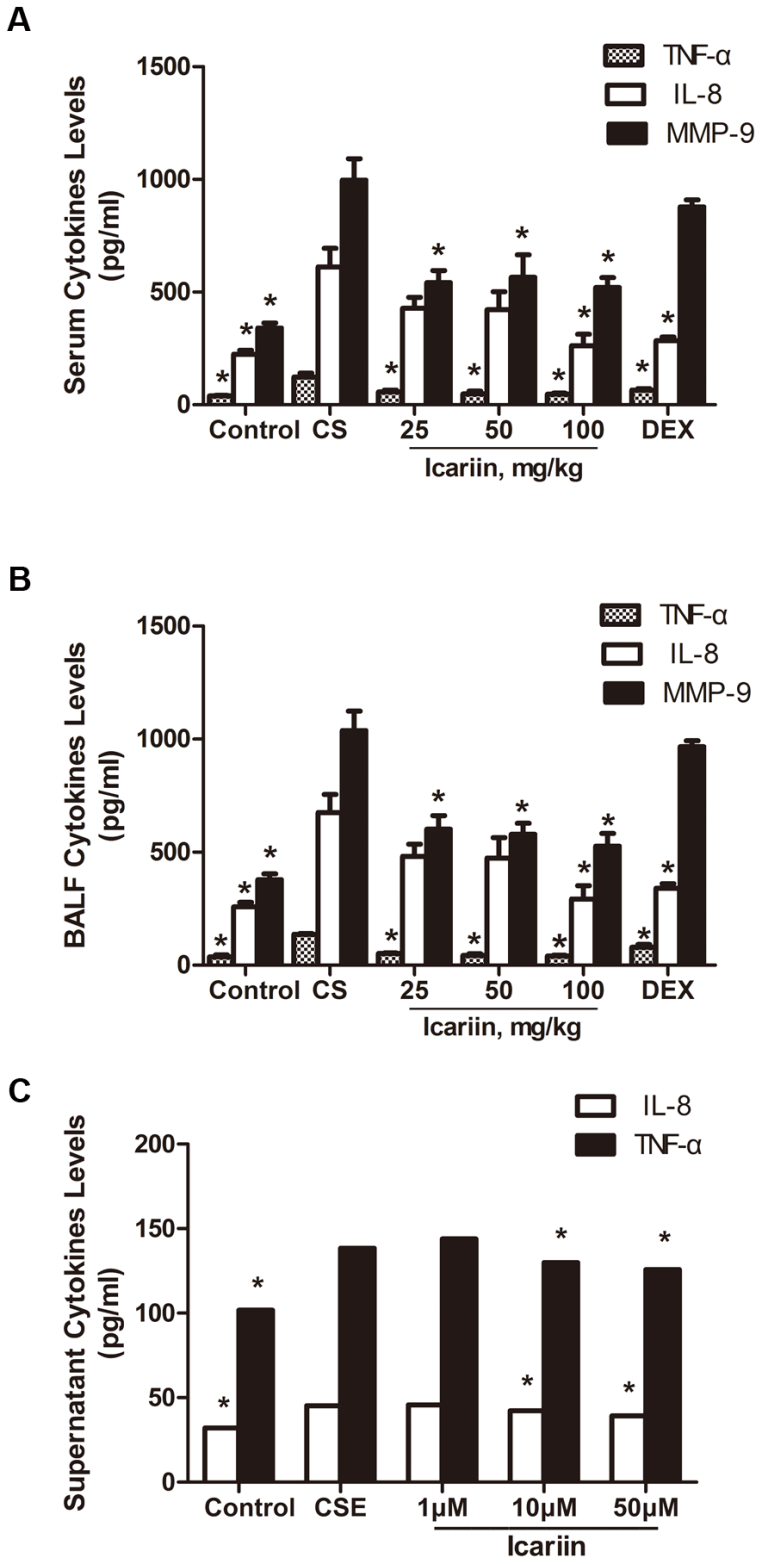
Icariin suppresses CS-induced TNF-α, IL-8 and MMP-9 levels *in vivo* and *in vitro*. TNF-α, IL-8 and MMP-9 levels in serum (A), BALF (B) and culture supernatant (C) were measured by ELISA. Data are mean ± SEM (n = 4–5). * P<0.05 vs. CS-exposed mice or CSE-exposed A549 cells. CS = cigarette smoke; CSE = cigarette smoke extract; DEX =  dexamethasone; BALF  = bronchoalveolar lavage.

To further characterize the effects of icariin on modulating inflammatory response to cigarette smoke exposure in mice lungs and supernatants of A549 cells, we analyzed some inflammatory cytokines. As shown in Fig. 4A and B, significant (P<0.05) increases of TNF-α, IL-8 and MMP-9 levels in both BALF and serum were observed following treatment with 3 months of CS-exposure compared with controls. Treatments with icariin (25 mg/kg, 50 mg/kg, 100 mg/kg) significantly (P<0.05) inhibited TNF-α, IL-8 and MMP-9 production in serum compared with CS-exposed mice. In contrast, dexamethasone significantly reduced production of TNF-α and IL-8 compared with CS-exposed mice, but failed to (P>0.05) suppress the elevated levels of MMP-9 (Fig. 4A). A similar response was also observed in BALF (Fig. 4B). Furthermore, our in vitro experiments also showed that middle and high concentrations of icariin (10 µM and 50 µM) obviously (P<0.05) suppressed CSE-elevated productions of IL-8 and TNF-α in supernatant of A549 cells (Fig. 4C).

### Effects of icariin on CS-induced NF-κB activation

**Figure 5:**
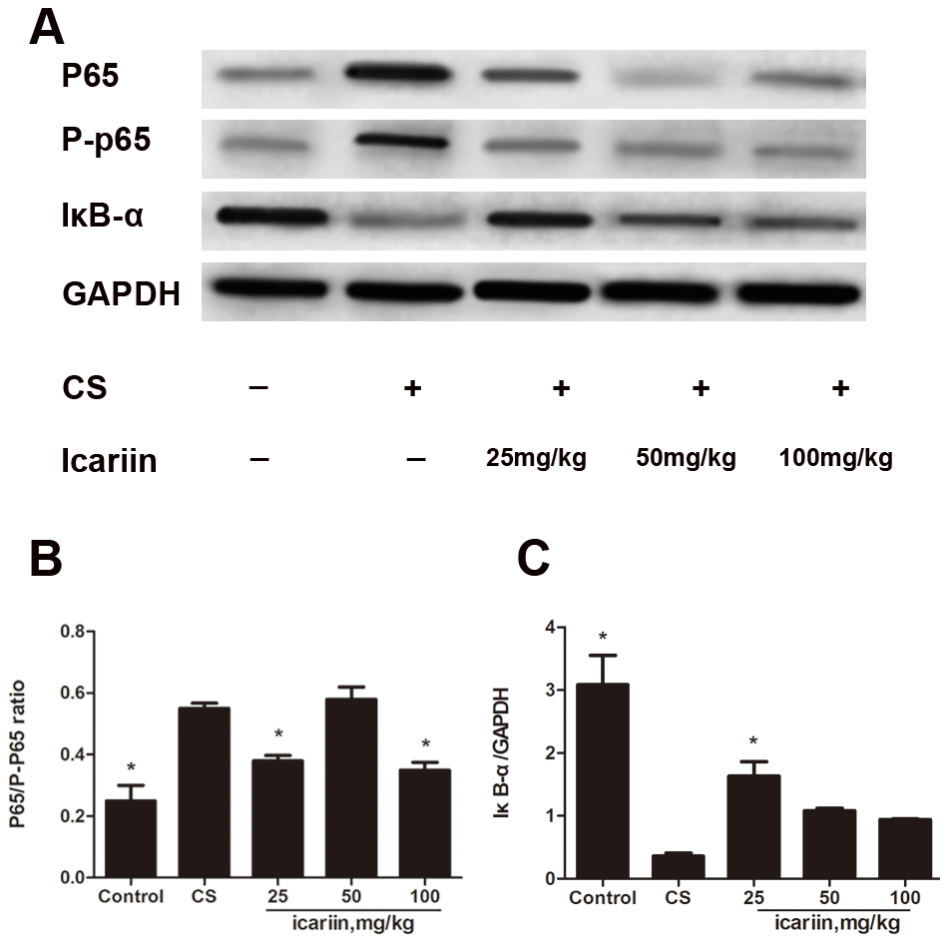
Icariin inhibits NF-κB activation in CS-induced inflammation. Mice were exposed to CS for 3 months and dosed with icariin (25, 50 and 100 mg/kg) or dexamethasone (1 mg/kg). (A) Nuclear proteins were extracted from lung tissue and p65, p-p65 and IκB-α protein expression was measured by Western blot. The phosphorylation of p65 (B) and the degradation of IκB-α (C) were calculated using gray value ratio of p65/p-p65 and IκB-α/GAPDH, respectively. Data are mean ± SEM (n = 3). * P<0.05 vs. CS-exposed mice.

Based on our previous findings that icariin inhibiting the LPS-induced NF-κB activation *in vivo* and *in vitro* [16], we also investigated whether icariin could interfere with the NF-κB activation in lungs of CS-exposed mice. As shown in Fig. 5, 3 months of CS-exposure markedly (P<0.05) increased the phosphorylation of nuclear NF-κB p65 and the degradation of IκB-α. Icariin apparently (P<0.05) inhibited the expression of p65 protein phosphorylation and blocked the degradation of IκB-α, indicating that icariin had inhibitory effect on CSE-activated NF-κB pathway.

### Effects of icariin on CS-mediated protein and mRNA expression of GR

**Figure 6:**
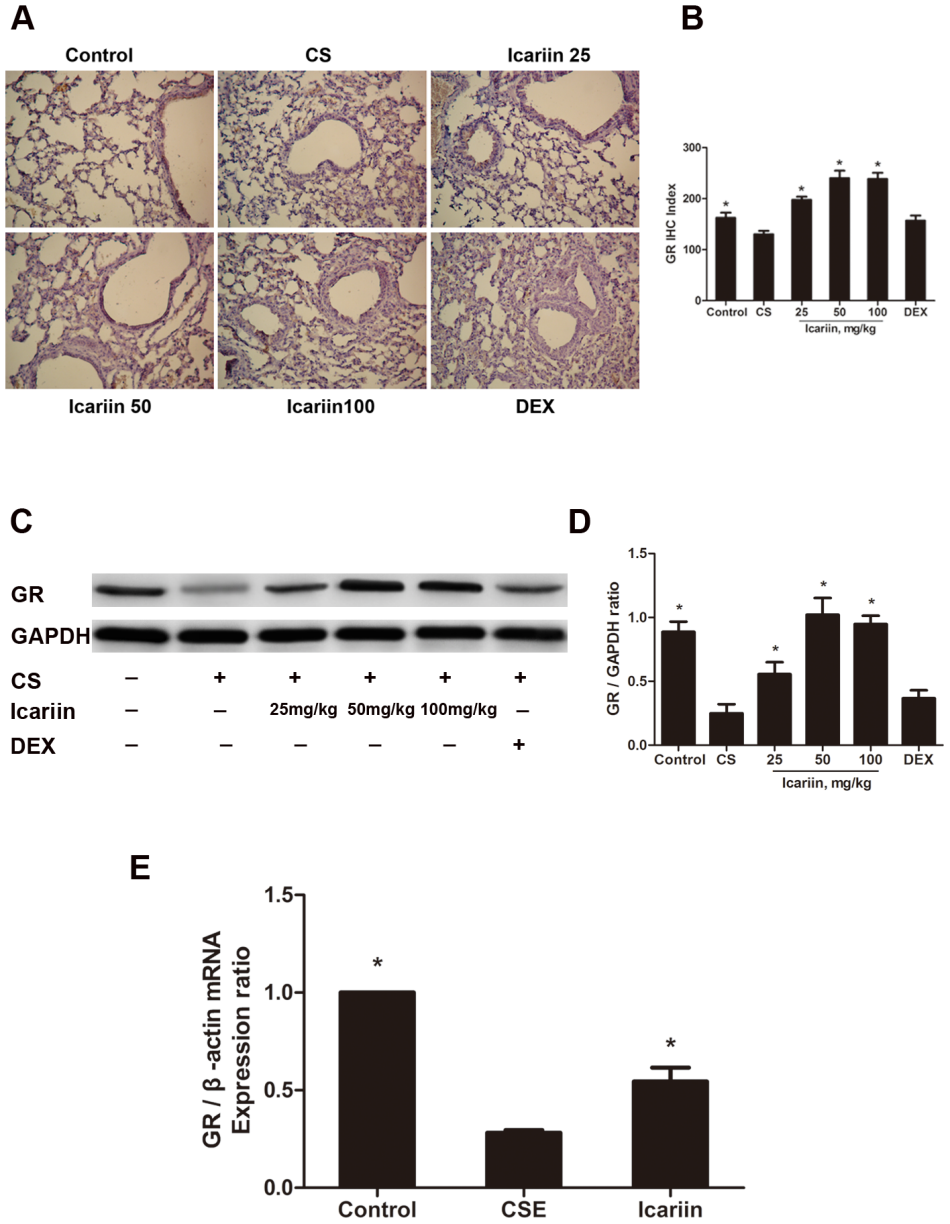
Icariin restores CS-mediated reduction in expression of GR protein and mRNA *in vivo* and *in vitro*. Nuclear proteins were extracted from lung tissue and GR protein expression was measured both by Immunohistochemistry and Western blot. (A) Micrographs of GR immunostaining in mice. (B) Immunohistochemical index of GR. Quantitative immunohistochemical evaluation values of GR were calculated as described in Materials and methods. Data are mean ± SEM (n = 6). (C, D) Protein expression of GR in lung tissue of mice treated with different ways using Western blot. Data are mean ± SEM (n = 3). (E) Icariin restores CSE-reduced expression of GR mRNA *in vitro.* Real-Time PCR were performed to assess the levels of mRNA expression of GR. Data are mean ± SEM (n = 3). * P<0.05 vs. CS-exposed mice or CSE-exposed cells. CS = Cigarette smoke; CSE = Cigarette smoke extract; DEX =  dexamethasone.

As GR has been shown to be an essential co-factor for the anti-inflammatory efficacy of corticosteroids, we investigated the impact of icariin on GR expression in mice lungs and in A549 cells. Immunohistochemical analysis revealed that GR IHC index decreased significantly in CS-exposed mice compared to that of the control and subsequent three doses of icariin treatments remarkably (P<0.05) enhanced CS-impaired GR IHC index (Fig.6A, B).

To verify the results obtained from Immunohistochemistry, we further examined the expression of GR using western blots. As expected (Fig.6C, D), CS-exposed mice at 3 months significantly showed decreased GR protein levels compared with air-exposed mice and icariin apparently blocked this CS-induced GR degradation. In contrast, there were no significant differences on GR protein level between dexamethasone group and CS-exposed group (P>0.05) (Fig.6 A–D), indicating that dexamethasone failed to restore the CS-impaired protein expression of GR and there did exit the corticosteroid insensitivity in CS-induced inflammatory mice model.

We next examined whether icariin also has a protective effect on GR as observed *in vivo*, by treating A549 cells with CSE (2.5%) or icariin (10 µM). Real-Time PCR were performed to assess the levels of mRNA expression of GR. Our results show that the expression of GR mRNA in CSE-exposed group was significantly (P<0.05) lower than that in normal control. However, pretreatment of A549 cells with icariin obviously (P<0.05) attenuated the CSE-mediated reduction in GR mRNA (Fig. 6E), suggesting that icariin-mediated increase in GR protein expression likely be due at least in part to enhancement in GR gene transcription, leading might to the inhibition of CS-induced inflammatory responses.

## Discussion

In the present study, we have demonstrated that icariin, an active component purified from the Chinese herbal plant *Epimedium*, displays obviously anti-inflammatory effect in cigarette smoke induced inflammatory models in mice and A549 cells. *In vitro*, pretreatment with icariin attenuated lung injury by protecting pulmonary function, suppressing the production of inflammatory mediators such as TNF-α, IL-8 and MMP-9, inhibiting NF-κB activation, as well as enhancing the GR expression, which might contribute to decreasing airway inflammatory response with corticosteroid sensitivity. *In vivo*, pretreatment with icariin suppressed the production of TNF-α and IL-8 as well as enhancing the GR mRNA expression.

Cigarette smoke, which induces oxidative stress to drive an inflammatory response, is considered to be the primary risk factor for development of COPD [28] and the major cause of corticosteroid resistance observed in patients with COPD [29]. The current hypothesis suggests that cigarette smoke leads to airway inflammation by activating macrophages, neutrophils and T lymphocytes, which release proteases and reactive oxygen species (ROS) causing cellular injury [30], [31]. As a consequence, oxidative stress are triggered to lead to the activation of redox-sensitive transcription factors, such as NF-κB and activator protein-1 (AP-1), which are critical to transcription of pro-inflammatory genes (IL-8, IL-6, and TNF-α) [32], [33].

For the study, COPD was induced by 3 months of cigarette smoke exposure in BALB/c mice. We observed characteristic pathological changes consistent with chronic bronchitis and emphysema which are characteristic of COPD, and found that icariin significantly decreased values of inflammatory scores, MLI and DI in CS-exposed mice, and inhibited the CS-induced production of TNF-α, IL-8 and MMP-9 in both BALF and serum, showing an obviously anti-inflammatory capacity in mice. Moreover, the reductions in PEF, PIF and MV in response to CS were elevated by icariin, supporting the property of icariin for reversing airway obstruction and subsequently protect pulmonary function. Collectively, our results suggest that icariin were effective in protecting pulmonary function and suppressing inflammatory response. It is worthy to mention that PEF, PIF and MV in dexamethasone group were reduced compared to controls, which was consistent with findings that dexamethasone group showed significant weight loss than controls (data not shown). The mechanism by which dexamethasone failed to prevent the decrease of pulmonary function remains unknown, but at least it indicated that dexamethasone was insensitive to CS exposure. Thus, these results indicated that dexamethasone failed to show a completely anti-inflammatory efficacy, subsequently resulting in reduced corticosteroid sensitivity in mice. Alveolar epithelial cells play an important role in the regulation of immune and inflammatory responses in the lung [34]. As a result we also studied inflammation in A549 epithelial cells in response to CSE. Our data from *in vitro* studies showed that icariin significantly decreased the CSE-induced production of TNF-α and IL-8.

In an inflammatory setting, NF-κB plays a critical role in the regulation of many genes which are responsible for the synthesis of cytokines, adhesion molecules, chemokines, growth factors and enzymes [35]. NF-κB-mediated gene expression is controlled by a range of different enzymatic signalling events at multiple levels. In COPD, GR is involved in suppressing transcription factors NF-κB-induced inflammatory gene expression in COPD, by forming a complex with NF-κB. This complex prevents the DNA binding, and thereby gene transcription, by NF-κB [36].

We had previous proved that icariin significantly inhibit the LPS-induced NF-κB activation *in vivo* and *in vitro*. Our findings that pretreatment with icariin diminished the elevated inflammatory response seen in mice lungs after 3 months of CS-exposure may be due to the inhibition of NF-κB. Thus, the effect of icariin on modulating NF-κB was further investigated. In the current study, we found a significant enhancement in IκB-α protein expression in response to icariin along with a decrease in p65 protein phosphorylation. These results suggest that the attenuation of NF-κB activation seen in the icariin-treated mice would contribute to controlling airway inflammation. It can be concluded that icariin has significant anti-inflammatory effects on CS induced COPD mice and CSE-induced cell models, and the anti-inflammatory effect is likely achieved by inhibiting the NF-kB pathway.

Impaired corticosteroid sensitivity has been observed in CS-induced inflammatory models and patients with COPD [8], [37]. GR, which is widely distributed in animal and human cells of airways [38], is considered to play an important role in glucocorticoid responsiveness. The link between impaired corticosteroid sensitivity and COPD is based on GR and NF-κB in the lung [10]. Previous findings show that GR expression was significantly lower in animals exposed to cigarette smoke and patients with COPD than that in normal control, leading to increased transcription of pro-inflammatory genes associated with a resultant amplification of the inflammatory development [39], [40].

Considering the important role GR plays in the regulation of inflammatory responses in the lung, we further studied the GR expression in response to CS using immunohistochemical analysis, western blots and Real-Time PCR *in vivo* and *in vitro*. In our experiment, 3 months of CS-exposure did reduce GR protein expression by immunohistochemical analysis and western blots, respectively, and 2.5% concentration of CSE-treatment did reduce GR mRNA expression by Real-Time PCR. We did not find that dexamethasone attenuated CS-induced down-regulation of GR compared with icariin, suggesting that dexamethasone failed to work on the inflammation in COPD mice, which was consistent with our previous data [41] and might partly account for the glucocorticoid insensitivity in the disease. However, our results demonstrated that icariin administration significantly restore CS-reduced GR protein and mRNA expression, which might subsequently contribute to the attenuation of CS-induced respiratory inflammatory response.

Patients with COPD often exhibit higher plasma concentrations of inflammatory mediators such as IL-8 and TNF-α than that of healthy people [3], [4]. Given that in an inflammatory setting, GR represses a large set of functionally related inflammatory response genes by disrupting p65-interferon regulatory factor complexes [42], inflammatory mediators and their signaling pathways may play key roles in altered GR function of major COPD. Our previous studies have shown that icariin displays effectively anti-inflammatory potential, which include inhibition of TNF-α and IL-6 in the serum, as well as down-regulation of the mRNA expression of TNF-α, IL-6, iNOS and COX-2 in the lung of LPS-challenged models *in vivo* and *in vitro* [16], [17]. Moreover, in the current study we further provide evidence that icariin exhibited potent anti-inflammatory effect in CS-induced inflammatory mice. Thus, we hypothesize that icariin induced GR up-regulation maybe partly due to its anti-inflammatory efficacy.

In conclusion, icariin has significant effects in ameliorating cigarette smoke induced inflammatory responses, showing the potential to become an effective treatment of COPD. Our data also provide further support for the critical role icariin plays in normalizing GR expression and repressing NF-κB, which may be a mechanism underlying the anti-inflammatory effect of icariin in CS-induced inflammation. In view of settings that GR is involved in suppressing NF-κB-induced inflammatory gene expression in COPD, by forming a complex with NF-κB, further studies are necessary to investigate the relation between NF-κB pathway and GR activation about icariin anti-inflammatory mechanism in COPD.
